# Multi-functionality Redefined with Colloidal Carotene Carbon Nanoparticles for Synchronized Chemical Imaging, Enriched Cellular Uptake and Therapy

**DOI:** 10.1038/srep29299

**Published:** 2016-07-11

**Authors:** Santosh K. Misra, Prabuddha Mukherjee, Huei-Huei Chang, Saumya Tiwari, Mark Gryka, Rohit Bhargava, Dipanjan Pan

**Affiliations:** 1 Department of Bioengineering, University of Illinois at Urbana-Champaign, Urbana, Illinois 61801, USA; 2 Electrical and Computer Engineering, Chemical and Biomolecular Engineering, Chemistry, and Mechanical Science and Engineering, Beckman Institute for Advanced Science and Technology, University of Illinois at Urbana-Champaign, Urbana, Illinois 61801, USA

## Abstract

Typically, multiplexing high nanoparticle uptake, imaging, and therapy requires careful integration of three different functions of a multiscale molecular-particle assembly. Here, we present a simpler approach to multiplexing by utilizing one component of the system for multiple functions. Specifically, we successfully synthesized and characterized colloidal carotene carbon nanoparticle (C^3^-NP), in which a single functional molecule served a threefold purpose. First, the presence of carotene moieties promoted the passage of the particle through the cell membrane and into the cells. Second, the ligand acted as a potent detrimental moiety for cancer cells and, finally, the ligands produced optical contrast for robust microscopic detection in complex cellular environments. In comparative tests, C^3^-NP were found to provide effective intracellular delivery that enables both robust detection at cellular and tissue level and presents significant therapeutic potential without altering the mechanism of intracellular action of β-carotene. Surface coating of C^3^ with phospholipid was used to generate C^3^-Lipocoat nanoparticles with further improved function and biocompatibility, paving the path to eventual *in vivo* studies.

Nanomedicine shows enormous promise for diverse biomedical fields such as diagnostic imaging^1^,^2^,^3^,^4^,^5^, therapy^6^ and image-guided therapies^7^,^8^. At a nanometer scale, the properties of agents can be precisely tuned with respect to their composition, size, shape and the functionalities at the surface^9^,^10^. In addition to individual properties, nanoparticle ‘multi-functionality’ is immensely attractive, providing impetus to develop specialized particles tailored for specific purposes. Typically, nanoparticles are the result of controlled self-assembly of various building blocks, which individually provide a specific desirable property to the final particles^11^,^12^,^13^. One of the most attractive properties has been prominent in preclinical studies, showing the benefits of using nanoparticulates as ‘excipients’ in reducing drug toxicity and efficacy. Despite the early promise, however, very few have actually been translated for human use due to a host of delivery, uptake and tracking issues^14^,^15^,^16^. The desire to enable nanomedicine solutions has prompted the design of “multifunctional” nanoparticles, where additional competences like targeting specific sites or function and image contrast enhancement are added. Introduction of multi-functionality can be defined with an extensive assortment of homing, imaging and agents leading to many applications, including imaging^5^,^17^,^18^ targeted therapy^19^,^20^ or imaging guided therapy^21^,^22^,^23^, sensing^24^,^25^, bio-separation^26^, cellular labeling^27^, and gene therapy^28^.

Although this approach is appealing, additional functionality typically requires additional synthetic steps and costs, more intricate characteristics and uncertain effects *in vivo* alongwith greater, unknown regulatory obstacles^29^. Balancing the trade-off between multi-functionality and complexity is the subject of significant ongoing research as projected clinical benefits will be highly reliant on the individual selections made and their cumulative effect when designing multifunctional nanoparticles. We particularly focus on the additional steps and protocols needed to synthesize multifunctional particles as the complexity and multicomponent characteristics of nanoparticles present a large number of supplementary variables that may extensively escalate the level of difficulty in regulating processes and predictability in a biological system^30^. We recognize that synchronous imaging, therapy and facilitated cellular uptake are the three key deliverables for particles that are most critical and would require three properties: enhanced particle transport through the cell membrane into the cells, inclusion of a potent ligand detrimental to cancer cells, and enhanced contrast for robust detection in complex cellular environments. Further, a single moiety-based particle can mitigate several foreseen complexities associated with clinical translation i.e. variable precursor and properties, engineering of a reproducible manufacturing process, assortment of orthogonal analytical methods for ample characterization, a satisfactory pharmacological and toxicity profile, and demonstration of safety and efficacy. Our design proposed in this study focuses on the use of lycopenes or carotenoid molecules for synergistically functioning in multiple roles. Carotenoids are typically a C-40 class of terpenoid-based antioxidants with unique optical absorptive properties^31^. It is believed that carotenoids arose early in evolution playing an essential role as membrane stabilizers^32^,^33^ and are known to appear in most of the cellular membranes. Their cellular location and orientation largely depends on their molecular structure, polarity, dielectric properties and alterations such as hydroxylation or esterification, presenting opportunities for engineering carotenoid-based agents with specific properties. Previous studies have demonstrated that β-carotene and other nonpolar carotenoids such as lycopene tend to adopt in parallel with the membrane surface but situate deep within the lipid hydrophobic center^34^,^35^.

Recognizing these properties, we hypothesized that nanoparticles carrying β-carotene functionality can be engineered to be efficiently taken up by cells compared to non-functionalized nanoparticles. Due to the presence of long chain conjugated alternating double bonds, further, the compound strongly absorbs in the visible region. Vibrational spectroscopic signals are also anticipated to be specific and large due to the presence of eight isoprene units, which are cyclized at each terminus. Owing to the presence of a large number of conjugated double bonds, β-carotene fluoresces in the visible and also exhibits intense Raman vibrational modes stemming from the backbone C=C vibrations^36^,^37^. This molecule has previously shown to exert various antioxidant^38^,^39^, anticancer^40^,^41^, and anti-metastatic^42^ effects in lung, breast and neuroblastoma cells. Hence, β-carotene presents a unique opportunity to use ‘multi’ dimensional properties of this single molecule. Carbon nanoparticles (CNP) synthesized from various sugar sources have been reported to exhibit remarkable optical properties such as broad band photoluminescence and intense vibrational Raman bands originating from graphitic modes rendering them easily detectable^24^. Figure 1A shows a hypothetical schematic portraying CNPs presenting carotenoids on the surface, inserted into the cellular phospholipid bilayer (Fig. 1B). Although many therapeutic materials have been previously incorporated within CNPs^8^,^28^, β-carotene incorporated carbon particles with multiplexing have remained largely unexplored. Many of the cancerous cells are known to be difficult for nanoparticle internalization and it makes therapy difficult through nano-delivery. To utilize the complete potential of a nanoparticle based therapeutic approach, a system can be designed equipped with an additional modality to improve cell internalization. We report passivating strategies for β-carotene on CNPs to produce colloidal carotene carbon nanoparticles (C^3^-NP) and their anti-proliferative effect on human cancer cells. Human melanoma (C32) and breast cancer (MDA-MB231 ER(-)) cells, were selected as model cell systems because nanoparticle internalization is difficult in these cell lines due to lack of prominent endocytotic path. C^3^-NP were traceable after their cellular internalization on incubation with cells in 2D cell culture and biological tissue (skin). For delivery to various cells nanoparticles with lipid membranes have been reported to play a very constructive role in cell membrane interaction^43^,^44^, mechanism of internalization^45^,^46^, and delivery of the cargo^47^. In line of such advantages, further, we evaluated the idea of coating C^3^-NP with phospholipids (Fig. 1C) using physico-chemical characterizations. We also evaluated the effect of this process in inhibiting growth of cancer cells.

## Results and Discussions

### Preparation of C^3^-NP and C^3^-Lipocoat

Carbon materials can be derived from macromolecules, whose well-defined compositions, structures, and topologies provide a background for assembly into nanostructures with controlled functional properties. Very recently we have reported an alternate, commercially amenable synthetic methodology to synthesize functionalized carbon nanospheres^8^. For the synthesis of C^3^ nanoparticles, a pre-passivation and *in situ* synthetic methodology was adopted following the latter route. A natural carbohydrate, nectar agave, was used as an inexpensive source of carbohydrates with surface pre-passivated with β-carotene. Linear polyethyleneglycol (mPEG; Mw = 5,000 Da) was considered for an *in situ* passivation of as-synthesized nucleating Carbon nanoparticles (CNPs) as a control nanoparticle (passivated). The synthesis of the pristine (bare, uncapped) CNP used no passivating agents. This synthetic procedure is cost-effective and involved only a simple hydrothermal step using a commercial hot plate. In a typical synthesis, commercial grade nectar agave (batch composition; 47–56% fructose and 16–20% glucose, rest is mixture of other sugars and water) was suspended with the passivating agent (desired wt.%), purged with argon and heated for 10–20 min. β-Carotene is a non-polar compound with high degree of conjugation. The compound was made soluble in ethanol and admixed with nectar agave in 1:1, 1:2, 3:1 and 3:4 mass ratio of agave nectar to β-carotene. Other ratios were also studied but resulted in as a poor colloidal dispersion, likely due to the inadequate *in situ* capping of the nucleating carbon core by β-carotene. Purified C^3^ nanoparticles were isolated from loosely bound β-carotene molecules following a repeated dispersion and centrifugation process. For coating C^3^ nanoparticles with phospholipids (C^3^-Lipocoat), a membrane freeze thaw co-sonication method was followed using lecithin-PC as an amphiphile. Produced nanoparticles were thoroughly characterized by multiple physico-chemical techniques to study their behavior in suspended and anhydrous form.

### Physico-chemical characterizations

**Figure 2:**
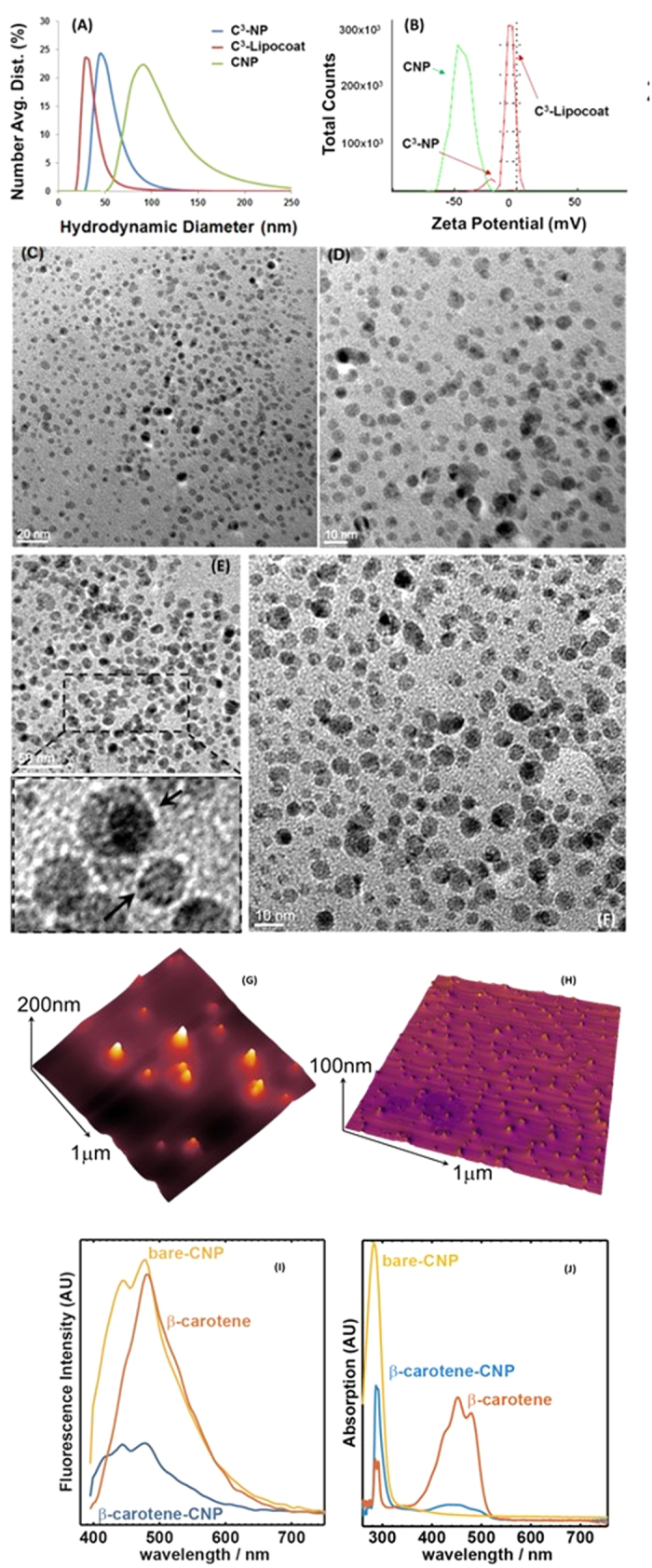
Physico-chemical characterization of C^3^-NPs and C^3^-Lipocoat nanoparticles. (**A**) Hydrodynamic diameter; (**B**) Zeta potential; anhydrous state size of (**C,D**) C^3^-NPs and (**E,F**) C^3^ Lipocoat nanoparticles by TEM; (**G,H**) height profile of C^3^-NPs by AFM. The spectroscopic evaluation of integral photonic properties of C^3^-NPs and compared to bare-CNP and β-carotene itself for (**I**) fluorescence properties and (**J**) UV-vis absorption efficiency. Properties were well compared with bare-CNP and β-carotene formulations to show the co-existence in C^3^ particles.

Figure 2A shows the hydrodynamic diameters of as-synthesized C^3^-NPs to be 44 ± 2 nm while bare-CNP was 97 ± 8 nm in size that further decreased to 25 ± 5 nm after phospholipid coatings. Likely unstable in an aqueous suspension, C^3^-NPs aggregate to bigger particles by bringing together other clusters of C^3^-NP in hydration state. On the other hand, we found that neither an increase nor a decrease in the relative mass ratio of agave nectar to β-carotene during preparation of particles generate a narrower size distribution. Thus we choose only the C^3^ nanoparticles prepared with a 1:1 mass ratio for further characterizations and biological studies. Pristine CNPs exhibited a shift of electrophoretic potential from −45 ± 5 mv to −20 ± 2 mv after being passivated with the hydrophobic nucleating agent β-carotene. Decrease in surface charge potential corroborates the ineptness of the C^3^-NPs to form stable aqueous suspensions compared to pristine CNPs. Furthermore, surface coating of phospholipid around the C^3^-NPs changes the zeta potential to −6 ± 1 mV (Fig. 2B) confirming the negatively charged nanoparticles. Although the charge potential decreased after phospholipid coating, C^3^-Lipocoat showed improved colloidal stability likely due to its enhanced degree of hydration. Anhydrous particles were studied by transmission electron microscopy (TEM) and atomic force microscopy (AFM). It showed a distribution of sizes for C^3^-NPs to be 32 ± 8 nm (Fig. 2C,D) very similar to the colloidal state diameter. The anhydrous sizes of phospholipid coated C^3^-Lipocoat NPs was found to be 20 ± 5 nm (Fig. 2E,F), slightly smaller than its hydrodynamic diameter of 25 ± 5 nm (Fig. 2A). The loss of hydration layer around lipid coating (Fig. 2G) while preparing TEM samples likely accounts for the slight decrease in size. Similar to the colloidal state, anhydrous C^3^-NPs were also slightly bigger than C^3^-Lipocoat nanoparticles. Probably, incorporation of phospholipid coating prevents C^3^-NPs to clump together giving smaller size on TEM sample preparation compared to more than one C^3^-NPs clumping together and giving a bigger size in absence of phospholipid coating (Table S1). AFM revealed the surface profile of semi-spheroidal structures from C^3^-NPs with height of 15 ± 5 nm (Fig. 2G,H). The ultraviolet-visible (UV-Vis) absorption spectrum of C^3^ and that of CNPs show strong absorption at ca. 300 nm which is the distinct signature of CNPs, whereas a weak, broad peak from 400–500 nm shown in C^3^ corresponding to characteristic of β-carotene (λ_max_ = 450 nm) (Fig. 2I). The fluorescence efficiency of CNPs were found to decrease on formulating to C^3^-NPs with β-carotene. (Fig. 2J). C^3^ have two prominent peaks roughly of the same intensity, one at 443 nm and the other at 479 nm; the former matches the second intense peak of CNPs, and the latter corresponds to the most intense peak of CNPs. Noticeably, the maximal fluorescence of β-carotene occurs at 483 nm, which likely contributes to the diminition of fluorescent intensity of C^3^ at 479 nm, bringing the peaks at 443 nm and 479 nm to similar intensity. One of the β-carotene absorption peaks at 283 nm was used to calculate the amount of β-carotene loaded onto C^3^ NPs instead of λ_max_ at 450 nm due to the strongly intense absorbance of CNPs, which made the difference in absorbance between CNPs and C^3^ indistinguishable in UV-Vis spectra. The overall loading percentages were found to be ~79% and used for the calculation of β-carotene concentration during various biological experiments. Colloidal stability of CNP, C^3^ and C^3^-Lipocoat NPs were studied by DLS measurement at various time points including 24, 48, 72, 96 and 120 h. It was found that CNP and C^3^-Lipocoat nanoparticles were stable without any significant variation in hydrodynamic diameter. Aqueous suspendability of CNP, C^3^ and C^3^-Lipocoat NPs was measured to be around 50, 10 and 60 mg/mL, respectably at ambient temperature. NPs were found to be stable for more than 6 months at this temperature. Suspendability of various nanoparticles in water was evaluated in comparative terms where C^3^ and C^3^-Lipocoat NPs were found to be highly dispersed in water compared to C^3^ NP (Table S1).

### Infra-red spectroscopic characterizations

**Figure 3:**
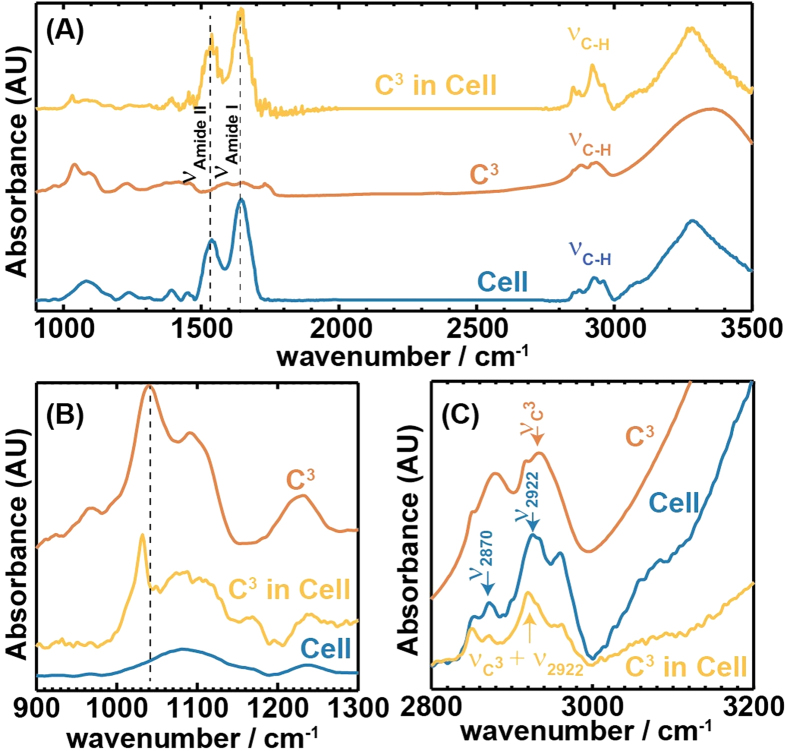
Characteristic Infra-red spectral features. Panel A shows the baseline corrected IR spectrum of a typical cell, C^3^ and C^3^ in cells. Spectra are offset for clarity. Panels B and C show the details of the IR spectra of the same in two different spectral regions. Characteristic spectral features of C^3^ are observed in cells as shown in Panel B at 1045 cm^−1^. Panel C displays the increase in the peak intensity ratio between ν_2922_ and ν_2957_ cm^−1^.

To structurally and spectroscopically characterize cells and tissue, we employ infrared (IR) spectroscopic imaging^48^,^49^. Shown in Fig. 3A are the average infrared spectra of cells (with no C^3^ incubation, control), C^3^, and cells incubated with a high concentration of C^3^ at 37 °C. Characteristic amide I and amide II bands are observed for both the controlled and incubated cells (blue and yellow). The fingerprint region of the C^3^ is broad and featureless except for a sharp peak at 1035 cm^−1^. Intense vibrational transitions for free β-carotene molecules are reported at ~966 cm^−1^ that arise from the deformation vibrations of the skeletal C-H bonds^50^. Weaker transitions are also observed at 1040 cm^−1^ that also results C-H deformation vibrations along with methylene (CH_2_) vibrations. The chemical nature of the passivation of the β-carotene molecules in the C^3^s suppresses these vibrational modes significantly (Fig. 3B) and intensifies the one at 1040 cm^−1^. An analysis of the infrared spectra of the cells incubated with high concentration of C^3^ revealed features (Fig. 3B yellow) that exhibit features similar to the C^3^ and cells taken together. However, the sharp feature of the C^3^ is red shifted to 1035 cm^−1^ due to binding with the cells. At low C^3^ concentrations and lower temperatures this characteristic peak is not observed in the IR spectra of the cells as the broad absorption of the phosphite (PO_2_^−^) peak and nucleic acids at 1080 cm^−1^ overwhelms any weak feature from the C^3^s in this region. Thus, we shift our focus to the C-H stretching region to assess the distribution of these particles over the cells at various conditions. In all the 3 spectra shown in Fig. 3C, we notice the presence of asymmetric CH_3_ (ν_2957_), CH_2_ (ν_2922_) and symmetric CH_3_ (ν_2870_), CH_2_ (ν_2850_) vibrations^51^. However, their peak ratios are remarkably different. For instance in the case of C^3^, the ratio of the asymmetric CH_2_ (~ν_2920_) stretch to symmetric CH_3_ (~ν_2870_) stretch modes is close to 1, while the same ratio is much greater than 1 for cells with or without the C^3^. We assign the main C^3^ peak at 2925 cm^−1^ to be originating from the C-H stretching vibrations of the C^3^ system (νC^3^) and its presence in cells will have an additional contribution at the same frequency. Thus, we expect the intensity ratio of the ν_2925_ band with any neighboring band will to increase when C^3^ is taken up by the cells.

**Figure 4:**
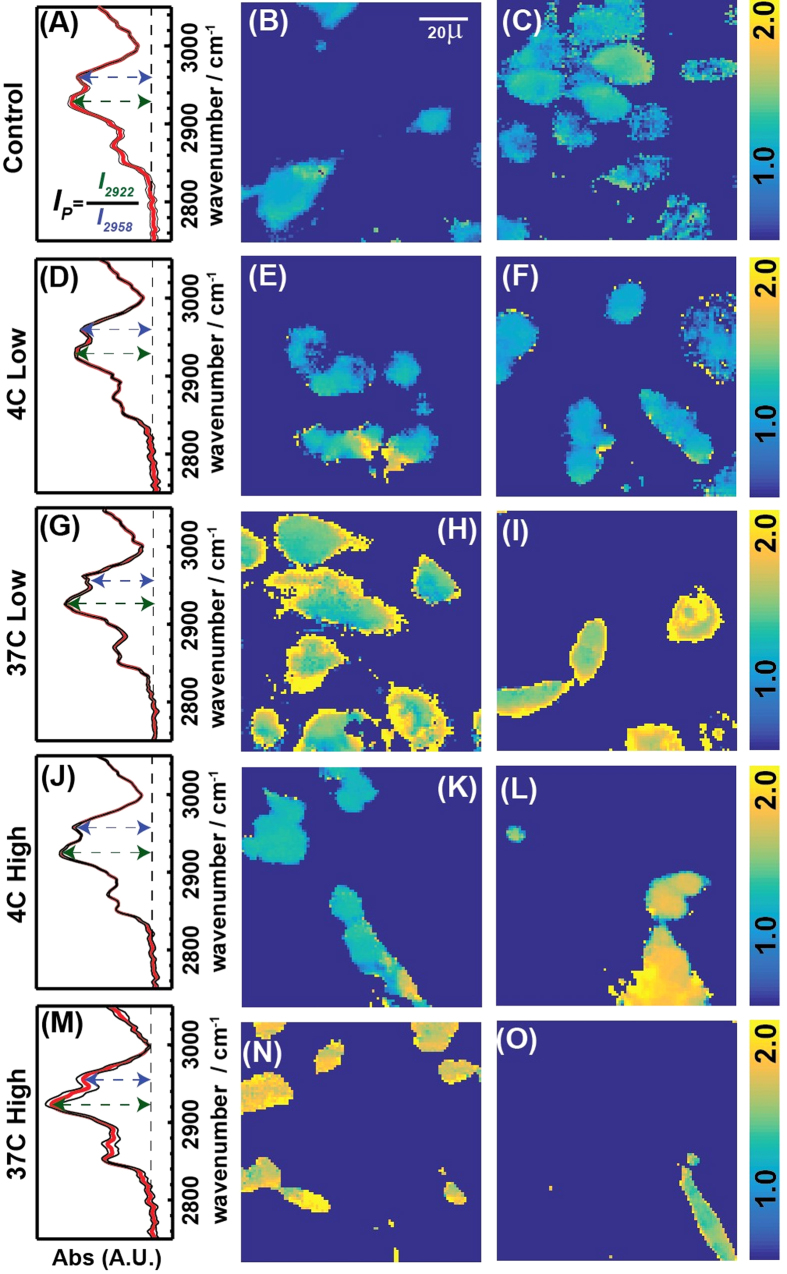
Infra-red cellular imaging. IR images of the cell and C^3^ incubated in cells for 5 cases as mentioned in the rows. (**A,D,G,J,M**) Show the average, baseline corrected IR spectra with the variance, while two separate images corresponding to each of the conditions are displayed in the rest. The intensity of each pixel is calculated as the ratio between 2922 and 2958 cm^−1^ band.

We calculated the pixel values of each of the IR images by taking the ratio of the band absorbance at 2922 and 2958 cm^−1^ from the spectrum of that pixel. Figure 4 shows the images of the absorbance ratio and the average IR spectra for five different conditions: (i) Cells without C^3^, (ii) Cells incubated with low concentration of C^3^ at 4 °C, (iii) same as the 2^nd^ condition but at 37 °C, (iv) Cells incubated with high concentration of C^3^ at 4 °C, (v) same as 4 but at 37 °C. The ratio distribution is almost homogeneous for cells without any C^3^ and rages from 1 to 1.2. In the 2^nd^ and the 4^th^ case when cells are incubated with C^3^ at 4 °C and 37 °C respectively, we observe higher ratio at the edges compared to the center. While this might arise from scattering, the close wavelengths used to calculate our ratio are unlikely to result in substantial scattering effects^52^,^53^,^54^ or band shifts. While the average intensity at the center of the cells in case 2 compares well with the cell image in case 1, case 4 shows a rise in the intensity even at the center of the cells. However, at 37 °C for both low and high concentrations of C^3^ (Case 3 and 5), we observe the absorbance ratio to rise to almost 2 both inside and at the cell edges. We attribute this change in intensity to the presence of the C^3^ taken up by the cells. β-carotene is known to be internalized by cells and express their therapeutic activities^38^,^39^,^40^,^41^,^42^ and likely facilitating the entry of carbon nanoparticles into the cells. At low temperature and lower concentrations they stick to the cell membranes as the uptake mechanism is not fully functional. But we do find a positive correlation between the uptake and concentration of C^3^ at low temperature. At 37 °C the cells uptake C^3^ readily and increasing the C^3^ concentration subjects the cell to perish faster as evident from the cell morphologies and cell viability studies.

### Raman spectroscopic characterizations

While infrared spectroscopy and imaging measure vibrational absorption^55^, Raman spectroscopy is a scattering process that depends on the polarizability tensor and is much weaker but is important to use here to understand the carbon skeletal contributions in the particles. Shown in Fig. S1A,B are the Raman spectra of cells (no C^3^), β-carotene (powder), C^3^ and C^3^ in cells. The wavelength axes are separated for clarity. Raman spectra of the cells by themselves are different from the IR spectra as the amide I and II modes collapse to an asymmetric broad band centered at 1650 cm^−1^. Strong modes at ~1450 cm^−1^ and 2923 cm^−1^ are observed that results from CH_2_ scissoring and asymmetric stretch vibrations. β-carotene displays 4 strong characteristic peaks at 1010 cm^−1^, 1185 cm^−1^, 1513 cm^−1^ and 3025 cm^−1^ resulting from ν(CH_3_), ν_C-C_, ν_C=C_ and ν_C-H_ vibrations respectively^51^.

C^3^ nanoparticles display two different kinds of Raman spectra depending on their concentration on coverslips. At low concentrations (green) they display three of the same features (ν (CH_3_), ν _C-C_, ν _C=C_) of that of β-carotene (blue) with a thermal oscillation. We find the ν _C=C_ mode in the C^3^ to redshift by 8 cm^−1^ to 1521 cm^−1^ that can be due to covalent interactions between the β-carotene and carbon nanoparticles. At higher concentrations the Raman spectra almost matches that of amorphous Carbon with characteristic G and D bands^56^ along with thermal oscillations. These oscillations are more evident in the 3 micron region of the spectrum where it overwhelms any C-H vibrational features (not shown for the aggregated C^3^). The excitation wavelength of the laser is set to 532 nm that is close to π−π^*^ transition of the carbon nanoparticles thereby triggering an almost electronic resonance condition and releasing heat, which affects the Raman spectra. If the excitation wavelength is changed to 633 nm or lower these oscillations are not observed.

**Figure 5:**
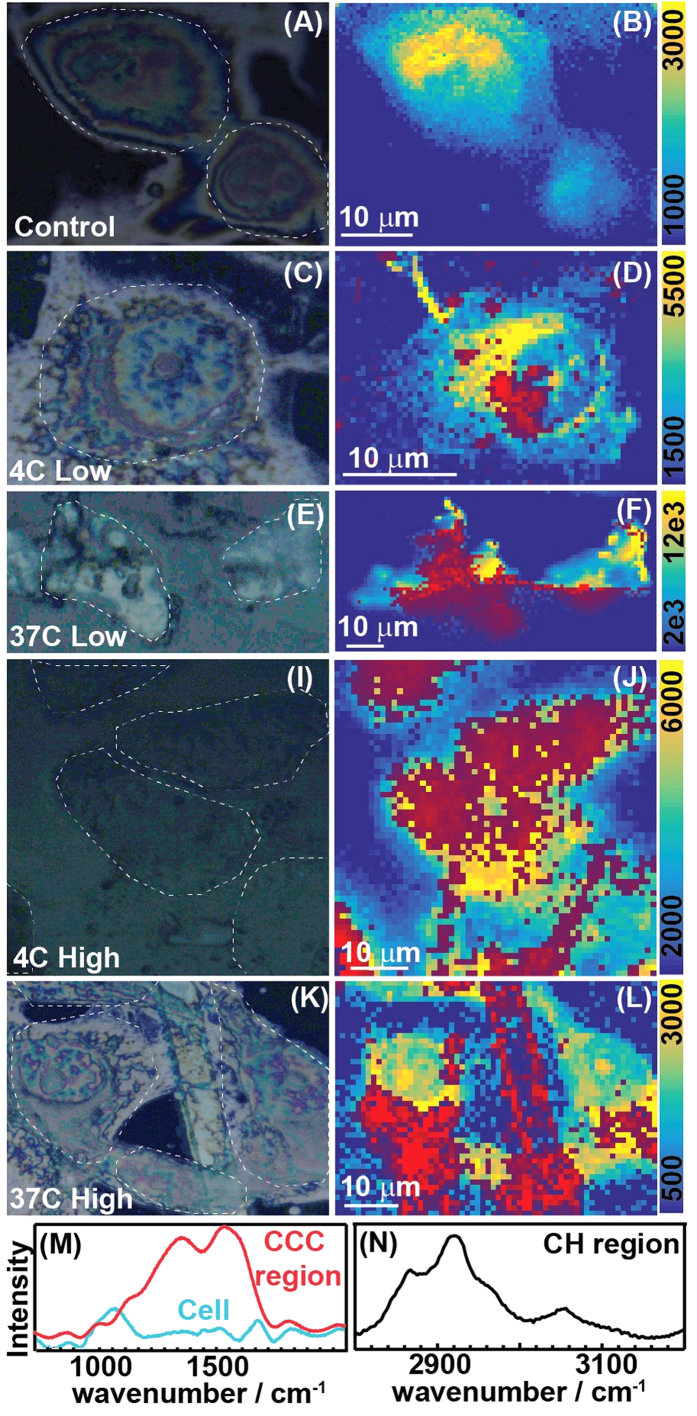
Raman cellular imaging. (**A,C,E,I,K**) Display the bright field images of breast cancer cells incubated with C^3^ at 5 different conditions. Raman images of the same regions are shown in (**B,D,F,J,L**) respectively. The C-H region intensity is plotted in the adjacent color bar while the C^3^ intensities are false-colored in red. Representative Raman spectra are also shown for cells only and cells incubated with C^3^ in M and N. Since the C-H region is not affected by C^3^, it is used to isolate cellular regions, while the Raman spectra of the CNPs are used to isolate the C^3^s.

Shown at the bottom of Fig. 5 (M and N) are representative Raman spectra of cells incubated with high concentration of C^3^. We observe that the spectrum exhibits similar features of cells (1650 cm^−1^, 1450 cm^−1^ and 2923 cm^−1^) and C^3^ ν (CH_3_), ν _C-C_, ν _C=C_). Since the presence of both the localized and aggregated C^3^ is evidenced through the Raman feature at 1520 cm^−1^ and cells from 2920 cm^−1^, we use these two bands to distinguish one from the other in the Raman images. We measured Raman images of cells under the same conditions as the IR studies. For each of these five cases, Raman images were constructed by calculating pixel intensities for each image in the following way. The 1^st^ image integrated the band intensities for the C-H vibrations and the 2^nd^ one integrated the C^3^ intensities. While the C-H intensities are plotted against a color bar, C^3^ intensities are false-colored in red on them. As expected the Raman image of the cells display no contribution from the C^3^s. The images for 4 °C show more uptake of the C^3^ inside the cells than the IR study. However, at 37 °C the red regions dominate the image showing the extent of their uptake by the cells. At higher concentrations we see the morphology of the cells to change with the presence of a channel of C^3^ particles between two cellular regions.

### *In vitro* cell studies

**Figure 6:**
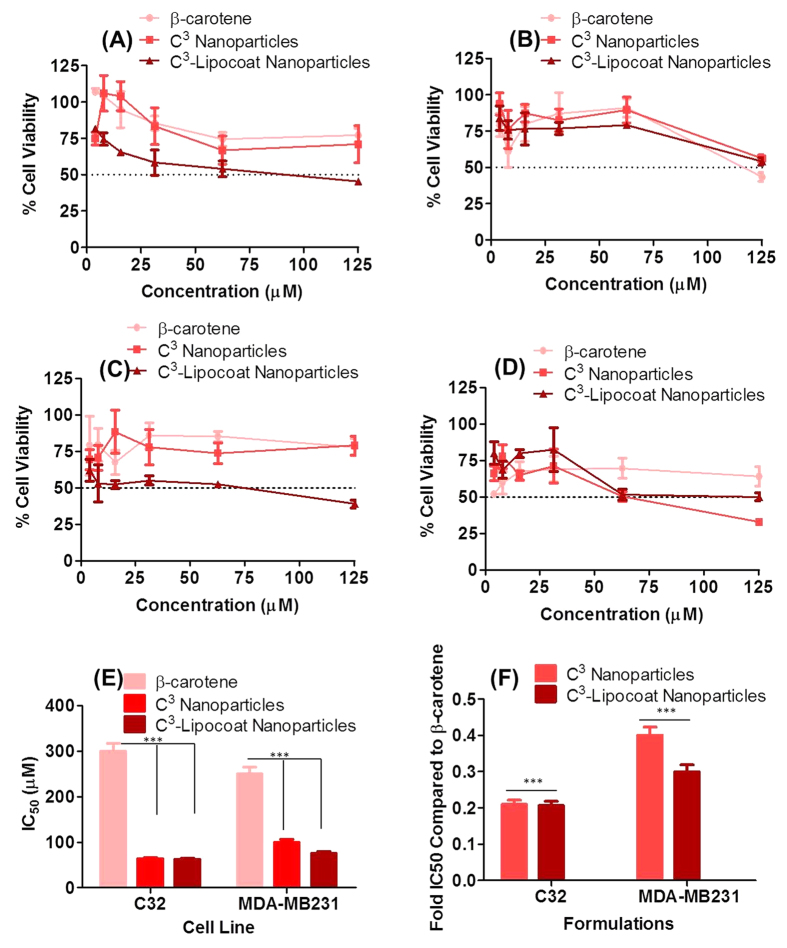
*In vitro* cellular studies. MTT assay for the evaluation of cytotoxic effects of β-carotene in free or passivated to CNPs with post coatings of amphiphilic phospholipidic assembly. Experiment was performed in (**A,C**) MDA-MB231 breast cancer and (**B,D**) C32 melanoma cells after incubation for (**A,B**) 48 and (**C,D**) 72 h at various concentrations (125, 62.5, 31.25, 15.6125, 7. 8 and 3.9 μM) of β-carotene in free or form of C^3^ nanoparticles and C^3^-Lipocoat nanoparticle formulations. (**E**) Comparative IC_50_ values of used formulations and (**F**) fold change across both the cell lines. Statistical analysis performed using Two-way ANOVA on IC50 values represented as *** for p < 0.001.

The cell growth inhibition property of C^3^ was evaluated against two human cancer cell lines C32 (melanoma) and MDA MB 231 (breast cancer) cells by a 3-(4, 5-dimethylthiazole-2-yl)-2, 5-diphenyltetrazolium bromide (MTT) assay (Fig. 6A–F). Cells were incubated with controls of CNPs, 1% of dimethyl sulfoxide (DMSO), β-carotene, C^3^ and C^3^-Lipocoat NPs for 48 and 72 h at β-carotene concentrations of 125, 62.5, 31.25, 15.6 and 7.8 μM and their equivalence in samples without β-carotene. It was found that neither CNPs nor 1% of DMSO induced significant cytotoxicity in any of the used cell lines at up to highest used concentration (not shown). The cell growth regression showed that order of efficiency followed the pattern of best to minimum efficiencies in C^3^-Lipocoat, C^3^ and β-carotene at 48 h of incubation (Fig. 6A,B). A significant decrease in cell viability was observed with IC_50_ = 300 ± 30 μM and 250 ± 25 μM in C32 and MDA MB 231 cells after 72 h treatment of β-carotene, respectively (Fig. 6C,D).

An improvement in IC_50_ was noticed after cell treatment in similar conditions with C^3^ nanoparticles at 72 h, which increased to 63 ± 06 μM and 100 ± 10 μM in C32 and MDA-MB231 cells, respectively. It was found to be further improved slightly by surface coating of C^3^ nanoparticles in lipid shell, namely the C^3^-Lipocoat particles. Incubation with C^3^-Lipocoat particles led to further decrease in IC_50_ values of 62 ± 06 and 75 ± 08 μM in C32 and MDA-MB231 cells, respectively after 72 h of incubation. Noticeably, both the cell lines responded to β-carotene, C^3^ and C^3^-Lipocoat after treating for 48 and 72 h time points. Two Way ANOVA was used to perform biostatistical analysis and found that C^3^ and C^3^-lipocoat NPs were significantly better in bringing down the IC50 values compared to β-carotene to a level of p < 0.001, irrespective of the cell line used.

To evaluate the effect of β-carotene formulations on non-tumorigenic epithelial cell line of mammary gland, MCF-10 A cells of human breast origin was used for MTT assay at much higher concentration of β-carotene in free or nanoparticulate form (C^3^-nanoparticles and C^3^-Lipocoat nanoparticles, 50–800 μM). Data showed that C^3^-nanoparticles and C^3^-Lipocoat nanoparticles were statistically less toxic to MCF-10 A cells compared to β-carotene, at all the used concentrations (Figure S2A). It implied that the better growth regression ability was shown by CNP formulations of β-carotene toward tumoregenic cells while less selectivity by β-carotene alone. None of the treated concentration of β-carotene formulations reached to growth regression of 50%. Integral absorbance from nanomaterials was studied at λ_592_ nm. The absorbance for formazan crystals was found to be very insignificant contributors to overall absorbance (Figure S2B) at this wavelength. All nanoparticles were used at concentration equivalent to highest used concentration of particles for MTT assay. All particles were dissolved in DMSO before acquiring the absorbance.

**Figure 7:**
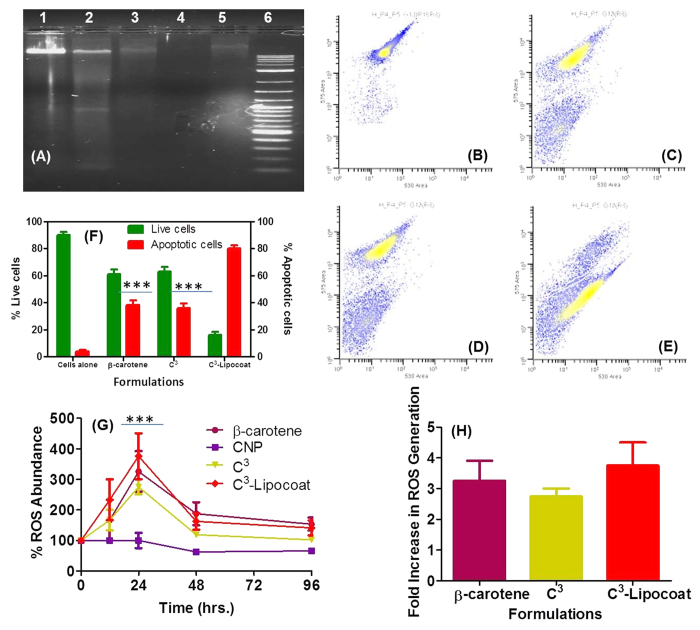
Mechanistic studies for induced apoptosis *via* reactive oxygen species (ROS) generation. (**A**) DNA laddering assay performed on genomic DNA extracted from MDA-MB231 cells (lane 1) untreated; or treated with (lane 2) C^3^-Lipocoat; (lane 3) C^3^ nanoparticles; (lane 4) CNP and (lane 5) β-carotene at concentration of 100 μM. Here lane 6 represent DNA ladder of 1–10 kb. Scatter plots obtained from 4% paraformaldehyde fixed MDA-MB231 cells (**B**) untreated or treated with (**C**) β-carotene; (**D**) C^3^ and (E) C^3^-Lipocoat at concentration of 100 μM post propidium iodide (PI; 10 μg/mL) staining. Scattering events in R7 and R2 gates represent the cells from live cell and apoptotic cell population, respectively. (F) Scattering events revealed the increasing % of apoptotic cell population in order of β-carotene, C^3^ and C^3^-Lipocoat while % live population followed the reverse order with maximum in untreated cells. (G) Variation in ROS abundance with time of incubation and change in β-carotene formulation in MDA-MB231 with maximum fold increase by C^3^-Lipocoat compared to CNP itself (H). Statistical analysis performed using Two-way ANOVA on IC50 values represented as *** for p < 0.001.

The functional activity of β-carotene in free or form of C^3^ and C^3^-Lipocoat was investigated by MTT assay and extended to mechanistic scrutiny for induced apoptosis by DNA laddering assay and PI mediated cell cycle analysis. The apoptotic cell death leads to fragmentation of genomic DNA into oligonucleosomal subunits *via* cell shrinkage and membrane blabbing. The genomic DNA results into approximately 200 bp sized pieces equivalent to nucleosomes and multiples thereof, generating 3′-OH groups at the strand breaks. DNA fragmentation was studied on 1% agarose gel using DNA laddering assay. It was performed on genomic DNA extracted from MDA-MB231 cells either untreated (Fig. 7A; lane 1) or treated with C^3^-Lipocoat (lane 2); C^3^ nanoparticles (lane 3); CNP (lane 4) and β-carotene (lane 5) at concentration of 100 μM. Produced DNA fragments in lane 2, and DNA smears in lane 3 and 5 revealed the induced apoptotic activity of cells treated with C^3^-Lipocoat, C^3^ nanoparticles and β-carotene in same order. A higher response of induced apoptosis in cells treated with C^3^, C^3^-Lipocoat NPs comapred to β-carotene probably signified the improved delivery of β-carotene in form of C^3^ and C^3^-Lipocoat NPs by controlling the interaction to cell membrane with CNP and Lipid coating, respectively. Probably, presence of CNP and Lipid coat also helped in further crossing over to cellular cytoplasm rather than holding in the membrane due to typical interaction patterns of β-carotene^32^,^33^. An improved C^3^-Lipocoat NPs response compared to C^3^, could be attributed to lipid coat further helping in delivering higer extent of β-carotene by improved particle suspendability. Induced apoptosis was further confirmed by PI intercalation in fragmented genomic DNA leading to low fluorescence cell population during cell cycle analysis. Cell population with lower PI staining, identified as sub-G0/G1 cell population, can be categorized as apoptotic cell population and directly correlates with ability to induction of apoptosis. PI staining was performed on MDA-MB231 cells after treatment with β-carotene (Fig. 7C), C^3^ nanoparticles (Fig. 7D) and C^3^-Lipocoat (Fig. 7E) for 72 h. The % apoptotic cell population was quantified as ~37, 37 and 80% cells for treatments with β-carotene, C^3^ nanoparticles and C^3^-Lipocoat, in same order (Fig. 7F). Improved apoptotic efficiency of β-carotene in form of C^3^-Lipocoat emphasized the probable role of lipocoat in improving β-carotene enrichment in treated cells (Table S2). Statistical analysis on apoptotic population induced by β-carotene, C^3^ and C^3^-Lipocoat NPs compared to untreated cells using Two way ANOVA revealed the significance of p < 0.001.

Human breast cancer cells (MDA-MB-231) are known to undergo induced apoptosis *via* increased production of reactive oxygen species (ROS)^57^. β-carotene is known to induce significant amount of ROS in breast cancer cells leading to cell apoptosis^58^. Induced apoptotic detection in MDA-MB231 cells treated with β-carotene and its CNP formulations C^3^ and C^3^-Lipocoat would indicate the plausible involvement of ROS generation leading to growth regression. To establish the involvement of ROS generation, a ROS detection assay was performed. MDA-MB231 cells were treated with 100 μM of β-carotene and its formulations as C^3^ and C^3^-Lipocoat along with control nanoparticles of CNP for different time points including 12, 24, 48, 72 and 96 h to measure the generation of ROS. A commercially available fluorometric intracellular ROS kit (Sigma-Aldrich; MAK143-KT) was used following manufacturers protocol on added fluorogenic sensor localized to the cytoplasm, resulting in a fluorometric product (λ_ex_ = 490/λ_em_ = 520 nm) proportional to the amount of ROS present. It was found that treatment with 100 μM of β-carotene and its formulations C^3^ and C^3^-Lipocoat, a significant level of ROS was generated within 12 h of treatment which remained elevated until 24 h post treatment. A reduction in the level of detectable ROS (Fig. 7G) was noticed thereafter, whereas pristine CNPs could not generate any significant level of ROS by itself. Quantification of produced ROS expressed the maximum ROS generation by C^3^-Lipocoat followed by C^3^ with not much difference from the case of β-carotene treatment (Fig. 7H). Possible proof of higher delivery efficiency of Lipocoat toward C^3^ nanoparticles could be improved abundance of β-carotene inside the cells which in turn produces more ROS. Statistical analysis on ROS generation level in cells treated with β-carotene compared to cells alone using Two way ANOVA revealed the significance of p < 0.001.

### *Ex-vivo* evaluation C^3^-NP for 3D imaging

**Figure 8:**
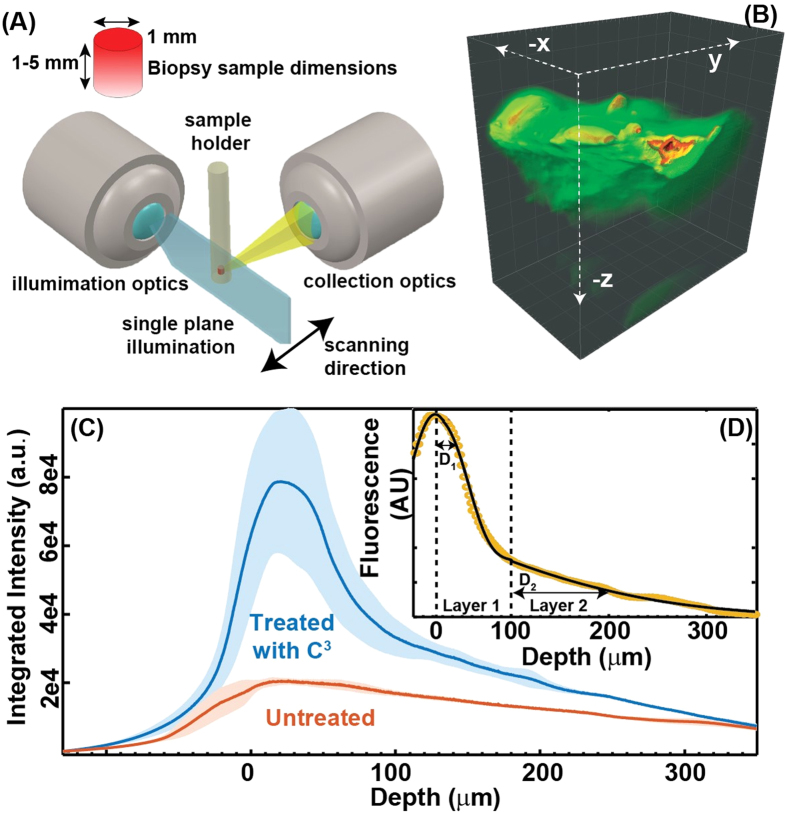
*Ex-vivo* fluorescence imaging. Panel A shows the 3D fluorescence experimental geometry. The objective generates a plane sheet of light that irradiates the sample. Fluorescence is acquired orthogonal to the light plane. Panel B shows a three dimensional rendering of background subtracted fluorescence images of pigskin treated with C^3^. Panel C shows the integrated fluorescence intensity with skin depth for both treated and untreated samples. Panel D shows the concentration profile of C^3^ within 2 layers of the skin upon diffusion.

3D imaging of the skin tissues with and without C^3^ incubated in them showed significant differences in the Fluorescence intensity distribution across the skin depth. Shown in Fig. 8A,B are the 3D rendering of the 500 2D fluorescence images of the C^3^ incubated pigskin and the bare skin respectively. The Z axis points to the direction from the surface into the skin. Maximum fluorescence intensities scattered from C^3^ incubated skin is an order more than that from the untreated skin. Integrating the pixel intensities from each of the 2D images along the skin depth we obtain the fluorescence profile shown in Fig. 8C for each of the two cases. The zero position (the skin surface) is assigned to the point where the average fluorescence is maximum and both of these profiles decay to a similar value at ~350 μm. Thus we attribute the increased fluorescence intensity at the skin surface to the presence of C^3^ and since the value decays to the same value as the untreated skin at the lower end we assume it to arise from the tissue itself. A simple subtraction of the averaged intensities from C^3^ treated skin and untreated skin results in the difference intensity profile shown in Fig. 8D. While spectroscopic imaging experiments into diffusion have also been conducted previously, the geometry here offers 3D tissue as well as rapid imaging with the high throughput imaging setup. Given the similarity in molecular diffusion between porcine and human skin^59^, we expect the results to translate well.

A simple one-dimensional two layer diffusion model was used to extract the diffusion depths in the two layers of the skin shown in the figure. The diffusion model is based on Fick’s second law where the concentration profile *C*(*x*,*t*) as the function of distance *x*, at a fixed time *t* is given by Equation 1.


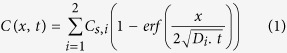


C_*s*,*i*_ being the concentration at the surface and the summation runs over 2 layers as described above, *D*_*i*_ being the diffusion coefficient for the *i*^*th*^ layer. Since the skin is not smooth and histologic cell types are not uniformly distributed in the skin the concentration at the surface is going to be defined by the skin roughness. We use a normal distribution to model it and use a Gaussian distribution for C_*s*,1_. From the figure we find that the first layer extends up to ~100 μm into the skin and the next layer starts thereafter, which correspond to the epidermis and hypo-dermis/dermis region of the skin respectively. We define the diffusion depth to be the point where the concentration is half of that at the beginning and that is estimated to be 25 μm for the first layer and ~100 μm for the second layer. The size of the nanoparticles enables them to diffuse into the skin more readily than other probes reported in the literature^60^. Furthermore, the distance of 100 mm for the second layer encompasses the epidermis. This means that these CNPs can readily facilitate drug unloading within the epidermis in ~10–20 hrs of their application.

## Conclusions

It is becoming increasingly vital to redefine multi-functionality of nanometer-sized particles for synchronous imaging, therapy and facilitated cellular uptake to provide the most effective relief. In this study we proposed a simple solution to the predicted complexities associated with clinical road blocks with respect to reproducibility, cost-effective manufacturing process, and assortment of orthogonal analytical methods for sufficient characterization, a satisfactory safety, toxicity and efficacy profile. Using a model system, we demonstrated that the design of this next generation particle can be envisioned by introducing a carefully selected single functional molecule to serve multiple purposes. We successfully synthesized and characterized β-carotene-decorated carbon nanoparticles and studied the feasibility of these particles to promote the intracellular delivery, contrast enhancement for robust detection at cellular and tissue level and therapeutic potential. As β-carotene is known to exert significant toxicity^61^, a nano-enabled delivery approach was highly desired to ensure controlled release. It was achieved by passivating β-carotene on CNPs. To ensure biocompatibility and systemic stability, a secondary phospholipids layer was post decorated on C^3^-NPs. Our results indicated that these particles can serve multiple functions where cancer cells can be targeted, measured and regressed. Although more experimental evidence will be required to determine whether these preliminary *in vitro* data are relevant to effects observed *in vivo*, our proposed solution of using simpler synthesis and single components to address multiple indications is a first step towards addressing many present limitations.

## Materials and Methods

Beta Carotene (β-carotene ≥95%, MW= 536.87 g/mol) was purchased from Cayman Chemical (Ann Arbor, MI, USA) and used without further purification. Beta Carotene is known to degrade photochemically. The stock reagent was protected from light and kept at 4 °C. Agave nectar (HoneyTree’s Organic Agave Nectar, MI, USA, 47–56% of fructose and 16–20% of glucose by weight with a remainder of other sugars and water) was obtained from a local grocery store. Lecithin-PC was purchased from NOF Corporation and used without further purification.

### Synthesis of colloidal beta-carotene carbon nanoparticles (C^3^)

A 1:1, 1:2, 3:1 and 3:4 mass ratio of agave nectar to β-carotene was dissolved in ethanol (200 proof, UPS specs, Decon Labs, Inc., PA, USA) and the aqueous solution was heated at 270 °C on a hot plate (Corning, Corning Life Sciences, MA, USA) for 30–40 min until the solvent evaporated. Followed by resuspension of C^3^ in ethyl ethanol and incubation overnight at room temperature, the sample was filtered by a *0.2-μm* pore filter (Millex, Merck Millipore Ltd., County Cork, Ireland) and incubated for at least 24 h to reach equilibrium prior to use.

### Preparation of C^3^-Lipocoat

Lipid coating of C^3^ nanoparticles was performed by freeze-thaw method. Briefly, soy lecithin (1 mg; 0.1 mg/mL) was solubilized in CHCl_3_ before evaporating it under rotary evaporator at 40 °C with reduced pressure. The prepared lipid membranes were kept under vacuum for >6 h to remove traces of organic solvent. These membranes were then hydrated with autoclaved water and C^3^-NPs for 12 h at 4 °C and were then processed through three cycles of thaw (5 min, 60 °C), freeze (5 min, 4 °C) and vortexing (2 min). After the sample was bath sonicated for 30 min to enable a tighter lipidic coating around C^3^-NPs, these particles were then purified by exhaustive dialysis against nanopure water (0.2 μm) using cellulosic membrane (10 kDa).

### Characterizations of C^3^

Hydrodynamic diameters and zeta potential of C^3^ were determined by a Malvern Zetasizer Nano ZS90 (Malvern Instruments Ltd., Worcestershire, UK) using water as a solvent. The averaged hydrodynamic diameter was obtained from the peak values of the intensity-weighted distribution with n≥3 and results were reported as mean ± standard deviation. Micrographs of transmission electron microscopy (TEM) was collected by JEOL 2010 LaB6 (JEOL Ltd., Tokyo, Japan) at an acceleration voltage of 200 kV. Samples were deposited on 200-mesh Quantifoil **holey carbon grids (**Structure Probe, Inc., PA, USA) to visualize their anhydrous sizes and morphology. Ultraviolet-visible (UV-Vis) absorbance of C^3^ and that of β-carotene were recorded via GENESYS 10S UV-Vis Spectrophotometer (Thermo Scientific, MA, USA). Absorbance spectra were collected at an interval of 1 nm from 250–900 nm. The emission spectra of fluorescence measurements were obtained through NanoDrop 3300 Fluorospectrometer (Thermo Scientific, MA, USA). The excitation maximum was set to 365 nm and the *wavelength* covered an excitation range between 400 and 750 nm.

### Cell Culture

C32 human *melanoma* and MDA MB 231 breast cancer *cell lines* were purchased from American Type Culture Collection (ATCC, VA, USA) and were cultured in minimum essential medium (MEM) and Dulbecco’s modification of Eagle’s medium (DMEM) (Corning Life Sciences, VA, USA), respectively, supplemented with 10% fetal bovine serum (FBS) and 1% penicillin-streptomycin (Gibco, Life Technologies, NY, USA).

### Cell viability assay

Cellular proliferation were evaluated by a 3-(4, 5-dimethylthiazole-2-yl)-2, 5-diphenyltetrazolium bromide (MTT) assay (Sigma-Aldrich, MO, USA). C32 or MDA MB 231 were plated on 96-well plates (Greiner Cellstar 96 well plates, Sigma-Aldrich, MO, USA) at a density of 1.0 × 10^4^ cells per well. After 24 h of incubation, cells were incubated with controls of CNPs and 1% of dimethyl sulfoxide (DMSO) and formulations of β-carotene without or with CNP (C^3^) and post lipidic coating in C^3^-Lipocoat for 48 and 72 h at concentrations of 125, 62.5, 31.25, 15.6 and 7.8 μM. Particles alone were used to collect blank readings at same concentration during analyzing the MTT assay results and deducted from the final values. After 44 or 68 h of incubation, 20 μl (5 mg/mL) of a MTT solution was added to each well and cells were further incubated for another 4 h. Media were aspirated and 200 μL of DMSO were added to dissolve formazan crystals produced. Absorbance was determined by Synergy HT (BioTek, USA) with a reference wavelength of 590 nm. The percentage of cell viability was determined via the following equation:


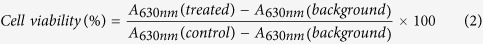


### MCF-10A cell culture and MTT assay for response to Non-tumorigenic mammalian cells

MCF-10A cells were thawed from frozen sample received from ATCC (ATCC CRL-10317) and was grown in 5% FBS containing MEGM medium (Kit Catalog No. CC-3150). 10,000 cells were plated in each well of 96 well plate 24 h before treating them with various formulations including β-carotene, C3-nanoparticles and C3-Lipocoat nanoparticles. After 24 h of incubation cells were incubated with formulations of β-carotene in free or form of C3-nanoparticles and C3-Lipocoat nanoparticles at concentration range of 50 to 800 μM for 72 h. At the end of incubation, MTT assay was performed as described for MDA-MB231 and C32 cells.

### Cell fixing for imaging and spectroscopy

For imaging and spectroscopic measurements we used human MDA-MB-231 breast cancer cells cultured in Dulbecco’s modified eagle’s medium (DMEM) supplemented with fetal bovine serum (FBS) and a mixture of Penicillin and Streptomycin. At 70% confluence, the cells were trypisinized and seeded onto sterilized low-E-slides (for infrared measurements) and coverslips (for Raman measurements). The cells cultured on both the low-E slides and coverslips were treated with the C^3^ nanoparticles for 6 hours at 37 °C and 4 °C. Cells were treated with low and high concentration of C^3^ Nanoparticles. Low concentration of C^3^ nanoparticles was fixed as concentration of IC50, whereas the high concentration of C^3^ nanoparticles were 4 times higher than the IC50 values. The IC50 value of the C^3^ nanoparticle was found to be ~200 μM. After incubation the cells were washed with phosphate buffer solution (PBS) and fixed using 70% ethanol. All the slides and coverslips were washed with deionized water to remove PBS from the cells before acquiring spectroscopic measurements.

### DNA laddering assay for evaluation of fragmented genomic DNA

MDA-MB231 (~0.3 × 10^6^) cells were grown in 6 well plates at ambient condition. After ~24 h of growing the cells were treated with 100 μM of β-carotene and its formulations as C^3^ and C^3^-Lipocoat for next 72 h. At the end of incubation period cells were trypsinized and collected in 400 μL of lysis buffer. The genomic DNA extraction was performed using manufacturer’s protocol using Thermo Scientific DNA extraction kit. Extracted genomic DNA were washed with 70% ethanol and dissolved in water after air drying.

### Propidium Iodide (PI) staining assay for measurement of apoptotic cell population

MDA-MB231 (~0.3 × 10^6^) cells were grown and treated as described in the DNA laddering assay experiments except at the end of incubation period, the cells were trypsinized and collected in 100 μl of reconstituted medium (DMEM containing 10% FBS) and fixed with chilled ethanol while vortexing. Fixed cells were stored at −20 °C for >12 h. At the end of the incubation, cells were washed with DPBS at least two times and incubated with RNase A (1 μg/mL) at 37 °C for >12 h. Cells were incubated with PI (2 μg/mL) for 30 min before scanning on FACS machine.

### Reactive Oxygen Species (ROS) generation assay

MDA-MB231 (~10 × 10^3^) cells were grown in 96 well plates at ambient condition. After 24 h of growth, cells were treated with 100 μM of β-carotene and its formulations as C^3^ and C^3^-Lipocoat along with control nanoparticles of CNP for different time points including 12, 24, 48, 72 and 96 h. Particles alone were used to collect blank readings at same concentration during analyzing the ROS assay results and deducted from the final values. ROS generation was measured using Fluorometric Intracellular ROS Kit (Sigma-Aldrich; MAK143-KT) following manufacturers protocol. ROS react with fluorogenic sensor localized to the cytoplasm, resulting in a fluorometric product (λ_ex_ = 490/λ_em_ = 520 nm) proportional to the amount of ROS present. Fluorescence was acquired at multi plate reader.

### Preparation of skin for 3D fluorescence depth of penetration studies

Freshly sacrificed pig’s feet were obtained from a local butcher and the skin was excised using a razor blade. The skin was then cut into one centimeter squares and fixed overnight in 4% paraformaldehyde at 4 °C. Fixed tissues were then washed three times in physiological buffer and clamped between a plain glass slide and a rubber spacer with circular hole. Nanoparticles were added to the rubber wells and allowed to diffuse for 16 hours in a humid environment. Post incubation, excess nanoparticle solution was aspirated and the tissues biopsied using 1 mm skin biopsy punches (Fisher Scientific). Biopsies were suspended in 1% agar in 1 ml syringes for light sheet imaging.

### Infrared spectroscopy

Infrared imaging of cells was performed in the attenuated total reflectance (ATR) mode on a Perkin Elmer Spotlight 400 system following the methods from literature^59^. All spectra were acquired using 4 cm^−1^ spectral and 1.56 μm spatial resolution. Eight scans were co-added for each pixel and the background was appropriately subtracted from each image.

### Raman spectroscopy

A high resolution research grade Horiba LabRAM HR Raman imaging system coupled to a Andor Newton back-illuminated EMCCD camera DU970P (1600 × 200 pixels and thermoelectric cooling to −70 °C) was used to acquire Raman spectra of the cells. The excitation wavelength was chosen to be 532 nm, 150 g/mm grating was used with the slit and confocal aperture fixed at 100 and 50 μm respectively. Each spectrum was acquired for 1 s with the laser power set to 25 mW. A 100x, NA 0.8 objective was used and the motorized XY stages were stepped 0.75 μm in both directions for imaging.

### Light Sheet-3D fluorescence imaging

Fluorescence depth measurements were acquired on agar embedded samples (Zeiss Z1 Lightsheet Microscope) using a 20x objective. Samples were excited using a 405 nm wavelength laser at 5 mW power and collected using an excitation filter. Depth measurements were taken over ~300 μm; exact measurement depth varied due to sample orientation. Samples were imaged over 250 planes with 1 μm slice thickness and an acquisition time of 200 ms. Three dimensional images were analyzed using Imaris 8.0 (Bitplane). 500 slices, each 250 nm apart, were analyzed as shown to assess nanoparticle penetration into the pig skin.

### Statistical analysis

To evaluate the significance of biological activity of C^3^ and C3-Lipocoat nanoparticles compared to β-carotene, statistical analysis was performed using Two way ANOVA followed by Bonferroni post test. Values of p were represented as *, ** and *** for p < 0.05, p < 0.005 and p < 0.001, respectively.

## Additional Information

**How to cite this article**: Misra, S. K. *et al*. Multi-functionality Redefined with Colloidal Carotene Carbon Nanoparticles for Synchronized Chemical Imaging, Enriched Cellular Uptake and Therapy. *Sci. Rep.* **6**, 29299; doi: 10.1038/srep29299 (2016).

## Supplementary Material

Supplementary Information

## Figures and Tables

**Figure 1:**
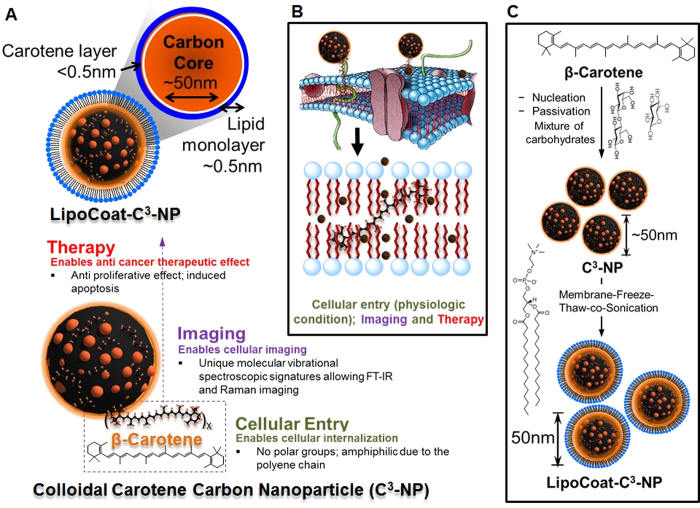
Schematic representation of C^3^-NP usases. (**A**) Graphical representation of a multifunctional nanoparticle system in anhydrous state presenting carotene functionalities on the surface for synchronous imaging, therapy and cellular transport. (**B**) Schematic portraying CNPs containing carotenoids driven into the cellular phospholipid bilayer. (**C**) Assembly of C^3^-NP from molecular to nanoscale, showing the final particle of use.

## References

[b1] Jia F. . Multifunctional nanoparticles for targeted delivery of immune activating and cancer therapeutic agents. J. Control. Release 172, 1020–1034 (2013). 24140748 10.1016/j.jconrel.2013.10.012

[b2] Sanvicens N. & Marco M. P. Multifunctional nanoparticles--properties and prospects for their use in human medicine. Trends Biotechnol. 26, 425–433 (2008). 18514941 10.1016/j.tibtech.2008.04.005

[b3] Bao G., Mitragotri S. & Tong S. Multifunctional nanoparticles for drug delivery and molecular imaging. Annu. Rev. Biomed. Eng. 15, 253–282 (2013). 23642243 10.1146/annurev-bioeng-071812-152409 PMC6186172

[b4] Lee D.-E. . Multifunctional nanoparticles for multimodal imaging and theragnosis. Chem. Soc. Rev. 41, 2656–2672 (2012). 22189429 10.1039/c2cs15261d

[b5] Misra S. K., Kim B., Chang H.-H. & Pan D. Defying symmetry: a ‘flowery’ architecture with augmented surface area from gold coated polymeric ‘nanoblooms’. RSC Adv. 4, 34303–34306 (2014).

[b6] Gindy M. E. & Prud’homme R. K. Multifunctional nanoparticles for imaging, delivery and targeting in cancer therapy. Expert Opin. Drug Deliv. 6, 865–878 (2009). 19637974 10.1517/17425240902932908

[b7] Smith L., Kuncic Z., (Ken) Ostrikov K. & Kumar S. Nanoparticles in Cancer Imaging and Therapy. J. Nanomater. 2012, 1 (2012).

[b8] Mukherjee P. . Tunable Luminescent Carbon Nanospheres with Well-Defined Nanoscale Chemistry for Synchronized Imaging and Therapy. Small 11, 4691–4703 (2015). 25994248 10.1002/smll.201500728

[b9] Albanese A., Tang P. S. & Chan W. C. W. The Effect of Nanoparticle Size, Shape, and Surface Chemistry on Biological Systems. Annual Review of Biomedical Engineering 14, 1–16 (2012). 10.1146/annurev-bioeng-071811-150124 22524388

[b10] Nikitin M. P., Zdobnova T. A., Lukash S. V., Stremovskiy O. A. & Deyev S. M. Protein-assisted self-assembly of multifunctional nanoparticles. Proc. Natl. Acad. Sci. USA 107, 5827–5832 (2010). 20231484 10.1073/pnas.1001142107 PMC2851910

[b11] Xiao Y. . Multifunctional unimolecular micelles for cancer-targeted drug delivery and positron emission tomography imaging. Biomaterials 33, 3071–3082 (2012). 22281424 10.1016/j.biomaterials.2011.12.030 PMC3313838

[b12] Misra S. K., Ye M., Kim S. & Pan D. Defined Nanoscale Chemistry Influences Delivery of Peptido-Toxins for Cancer Therapy. PLoS One 10, e0125908 (2015). 26030072 10.1371/journal.pone.0125908 PMC4452514

[b13] Misra S. K., Jensen T. W. & Pan D. Enriched inhibition of cancer and stem-like cancer cells via STAT-3 modulating niclocelles. Nanoscale 7, 7127–7132 (2015). 25785368 10.1039/c5nr00403a

[b14] Misra S. K., Kus J., Kim S. & Pan D. Nanoscopic poly-DNA-cleaver for breast cancer regression with induced oxidative damage. Mol. Pharm. 11, 4218–4227 (2014). 25140389 10.1021/mp500444c

[b15] Misra S. K., Ye M., Kim S. & Pan D. Highly efficient anti-cancer therapy using scorpion ‘NanoVenin’. Chem. Commun. (Camb). 50, 13220–13223 (2014). 25061638 10.1039/c4cc04748f

[b16] Kiessling F., Mertens M. E., Grimm J. & Lammers T. Nanoparticles for Imaging: Top or Flop? Radiology. 273, 10–28 (2014). 25247562 10.1148/radiol.14131520 PMC4186876

[b17] Lee K. J., Nallathamby P. D., Browning L. M., Osgood C. J. & Xu X.-H. N. *In Vivo* Imaging of Transport and Biocompatibility of Single Silver Nanoparticles in Early Development of Zebrafish Embryos. ACS Nano 2007, 1, 133. 10.1021/nn700048y PMC2613370 19122772

[b18] Altinoğlu E. I. . Near-Infrared Emitting Fluorophore-Doped Calcium Phosphate Nanoparticles for *In Vivo* Imaging of Human Breast Cancer. ACS Nano 2, 2075–2084 (2008). 19206454 10.1021/nn800448r

[b19] Patra C. R., Bhattacharya R., Mukhopadhyay D. & Mukherjee P. Fabrication of Gold Nanoparticles for targeted therapy in pancreatic cancer. Adv. Drug Deliv. Rev. 62, 346–361 (2010). 19914317 10.1016/j.addr.2009.11.007 PMC2827658

[b20] Bhattacharya R. . Attaching folic acid on gold nanoparticles using noncovalent interaction via different polyethylene glycol backbones and targeting of cancer cells. Nanomedicine Nanotechnology, Biol. Med. 3, 224–238 (2007).

[b21] Deng L. . Two-Step” Raman Imaging Technique To Guide Chemo-Photothermal Cancer Therapy. Chemistry 10.1002/chem.201502522 (2015). 26275063

[b22] Yang K. . Multimodal imaging guided photothermal therapy using functionalized graphene nanosheets anchored with magnetic nanoparticles. Adv. Mater. 24, 1868–1872 (2012). 22378564 10.1002/adma.201104964

[b23] Miller-Kleinhenz J. M., Bozeman E. N. & Yang L. Targeted nanoparticles for image-guided treatment of triple-negative breast cancer: clinical significance and technological advances. Wiley Interdiscip. Rev. Nanomed. Nanobiotechnol. 7, 797–816 (2015). 25966677 10.1002/wnan.1343 PMC4604004

[b24] Anker J. N. . Biosensing with plasmonic nanosensors. Nat. Mater. 7, 442–453 (2008). 18497851 10.1038/nmat2162

[b25] Misra S. K., Kim B., Kolmodin N. J. & Pan D. A dual strategy for sensing metals with a nano ‘pincer’ scavenger for *in vitro* diagnostics and detection of liver diseases from blood samples. Colloids Surf. B. Biointerfaces 126, 444–451 (2015). 25595484 10.1016/j.colsurfb.2014.12.048

[b26] Smith J. E., Wang L. & Tan W. Bioconjugated silica-coated nanoparticles for bioseparation and bioanalysis. TrAC Trends Anal. Chem. 25, 848–855 (2006).

[b27] Wei L. . Fabrication of bright and small size semiconducting polymer nanoparticles for cellular labelling and single particle tracking. Nanoscale 6, 11351–11358 (2014). 25141182 10.1039/c4nr03293d

[b28] Misra S. K., Ohoka A., Kolmodin N. J. & Pan D. Next generation carbon nanoparticles for efficient gene therapy. Mol. Pharm. 12, 375–385 (2015). 25514468 10.1021/mp500742y

[b29] Netti P. A., Berk D. A.,. Swartz M. A., Grodzinsky A. J. & Jain R. K. Role of extracellular matrix assembly in interstitial transport in solid tumors. Cancer Res. 60, 2497–2503 (2000). 10811131

[b30] Pardridge W. M. Adv. Drug Deliv. Rev. 1995, 15, 5. 35524389

[b31] Grassmann J. Terpenoids as plant antioxidants. Vitam. Horm. 72, 505–535 (2005). 16492481 10.1016/S0083-6729(05)72015-X

[b32] An G.-H., Suh O.-S., Kwon H.-C., Kim K. & Johnson E. A. Quantification of carotenoids in cells of Phaffia rhodozyma by autofluorescence. Biotechnol. Lett. 22, 1031–1034 (2000).

[b33] Gruszecki W. I. Carotenoids in membranes ; Frank H. A., Young A. J., Britton G., Cogdell R. J. Eds. Kluwer Academic Publishers, Dordrecht, 1999.

[b34] Kutuzov N. P. . Orientational ordering of carotenoids in myelin membranes resolved by polarized Raman microspectroscopy. Biophys. J. 2014, 107, 891. 10.1016/j.bpj.2014.07.002 PMC4142252 25140424

[b35] Van de Ven M., Kattenberg M., Van Ginkel G. & Levine Y. K. Study of the orientational ordering of carotenoids in lipid bilayers by resonance-Raman spectroscopy. Biophys. J. 45, 1203–1209 (1984). 6743750 10.1016/S0006-3495(84)84269-1 PMC1435001

[b36] Hoskins L. C. Resonance Raman spectroscopy of beta-carotene and lycopene, a physical chemistry experiment. J. Chem. Educ. 61, 460 (1984).

[b37] Rimai L., Heyde M. E. & Gill D. Vibrational spectra of some carotenoids and related linear polyenes. Raman spectroscopic study. J. Am. Chem. Soc. 95, 4493–4501 (1973). 4782645 10.1021/ja00795a005

[b38] Paiva S. A. & Russell R. M. Beta-carotene and other carotenoids as antioxidants. J. Am. Coll. Nutr. 18, 426–433 (1999). 10511324 10.1080/07315724.1999.10718880

[b39] Cerezo J. . Antioxidant properties of β-carotene isomers and their role in photosystems: insights from Ab initio simulations. J. Phys. Chem. A 116, 3498–3506 (2012). 22404131 10.1021/jp301485k

[b40] Gyémánt N. . Reversal of multidrug resistance of cancer cells *in vitro*: modification of drug resistance by selected carotenoids. Anticancer Res. 26, 367–374 (2006). 16475720

[b41] Palozza P. . Effect of beta-carotene-rich tomato lycopene beta-cyclase (tlcy-b) on cell growth inhibition in HT-29 colon adenocarcinoma cells. Br. J. Nutr. 102, 207–214 (2009). 19105854 10.1017/S0007114508169902

[b42] Lim J. Y., Kim Y.-S., Kim K.-M., Min S. J. & Kim Y. Β-carotene inhibits neuroblastoma tumorigenesis by regulating cell differentiation and cancer cell stemness. Biochem. Biophys. Res. Commun. 450, 1475–1480 (2014). 25019987 10.1016/j.bbrc.2014.07.021

[b43] Peetla C., Stine A. & Labhasetwar V. Biophysical interactions with model lipid membranes: applications in drug discovery and drug delivery. Mol. Pharm. 6, 1264–1276 (2009). 19432455 10.1021/mp9000662 PMC2757518

[b44] Roiter Y. . Interaction of nanoparticles with lipid membrane. Nano Lett. 8, 941–944 (2008). 18254602 10.1021/nl080080l

[b45] Ruiz de Garibay A. P., Solinís Aspiazu M. Á., Rodríguez Gascón A., Ganjian H. & Fuchs R. Role of endocytic uptake in transfection efficiency of solid lipid nanoparticles-based nonviral vectors. J. Gene Med. 15, 427–440 (2013). 24339018 10.1002/jgm.2749

[b46] Verma A. . Surface-structure-regulated cell-membrane penetration by monolayer-protected nanoparticles. Nat. Mater. 7, 588–595 (2008). 18500347 10.1038/nmat2202 PMC2684029

[b47] Muller R., Mäder K. & Gohla S. Solid lipid nanoparticles (SLN) for controlled drug delivery - a review of the state of the art. Eur. J. Pharm. Biopharm. 50, 161–177 (2000). 10840199 10.1016/s0939-6411(00)00087-4

[b48] Walsh M. J., Holton S. E., Kajdacsy-Balla A. & Bhargava R. Attenuated total reflectance Fourier-transform infrared spectroscopic imaging for breast histopathology. Vib. Spectrosc. 60, 23–28 (2012). 22773893 10.1016/j.vibspec.2012.01.010 PMC3388548

[b49] Walsh M. J., Reddy R. K. & Bhargava R. Label-free biomedical imaging with mid-infrared microspectroscopy. IEEE J. Sel. Top. Quant. 18, 1502–1513 (2012).

[b50] Berezin K. V. & Nechaev V. V. Calculation of the IR Spectrum and the Molecular Structure of β-Carotene. J. Appl. Spectrosc. 72, 157–161 (2005).

[b51] Schlucker S., Szeghalmi A., Schmitt M., Popp J. & Kiefer W. Structural analysis of cytochromes P450 shows differences in flexibility of heme 2- and 4-vinyls. J. Raman Spectrosc. 34, 413–419 (2003).

[b52] Davis B. J., Carney P. S. & Bhargava R. Theory of mid-infrared absorption microspectroscopy: II. Heterogeneous samples. Anal. Chem. 82, 3487–3499 (2010). 20392064 10.1021/ac902068e

[b53] Davis B. J., Carney P. S. & Bhargava R. Theory of midinfrared absorption microspectroscopy: I. Homogeneous samples. Anal. Chem. 82, 3474–3486 (2010). 20392063 10.1021/ac902067p

[b54] Mayerich D. . On the importance of image formation optics in the design of infrared spectroscopic imaging systems. Analyst. 139, 4031–4036 (2014). 24936526 10.1039/c3an01687k PMC4104806

[b55] Bhargava R. Infrared spectroscopic imaging: the next generation. Appl. Spectrosc. 66, 1091–1120 (2012). 23031693 10.1366/12-06801 PMC3756188

[b56] Robertson J. Diamond-like amorphous carbon. Mater. Sci. Eng. R Reports 37, 129–281 (2002).

[b57] Xie J. . Berberine-induced apoptosis in human breast cancer cells is mediated by reactive oxygen species generation and mitochondrial-related apoptotic pathway. Tumour Biol. 36, 1279–1288 (2015). 25352028 10.1007/s13277-014-2754-7

[b58] Cui Y. . beta-Carotene induces apoptosis and up-regulates peroxisome proliferator-activated receptor gamma expression and reactive oxygen species production in MCF-7 cancer cells. Eur. J. Cancer 43, 2590–2601 (2007). 17911009 10.1016/j.ejca.2007.08.015

[b59] Kong R. & Bhargava R. Characterization of porcine skin as a model for human skin studies using infrared spectroscopic imaging. Analyst 136, 2359–2366 (2011). 21509377 10.1039/c1an15111h

[b60] Desai P., Patlolla R. R. & Singh M. Interaction of nanoparticles and cell-penetrating peptides with skin for transdermal drug delivery. Mol. Membr. Biol. 27, 247–259 (2010). 21028936 10.3109/09687688.2010.522203 PMC3061229

[b61] Orazizadeh M., Khorsandi L., Absalan F., Hashemitabar M. & Daneshi E. Effect of beta-carotene on titanium oxide nanoparticles-induced testicular toxicity in mice. J. Assist. Reprod. Genet. 31, 561–568 (2014). 24515782 10.1007/s10815-014-0184-5 PMC4016370

