# Supplementary material for: Multi-functionality Redefined with Colloidal Carotene Carbon Nanoparticles for Synchronized Chemical Imaging, Enriched Cellular Uptake and Therapy
Source: Sci Rep. 2016 Jul 11;6:29299. doi: 10.1038/srep29299 (PMC4941412; doi:10.1038/srep29299)
Supplement: Supplementary Information [file srep29299-s1.doc]

**Supporting Information**

**Multi-functionality Redefined with Colloidal Carotene Carbon Nanoparticles for Synchronized Chemical Imaging, Enriched Cellular Uptake and Therapy**

Santosh K Misra,^a,†^ Prabuddha Mukherjee,^b, †^ Huei-Huei Chang,^a^ Saumya Tiwari,^b^ Mark Gryka,^b^ Rohit Bhargava,*^,b^ and Dipanjan Pan*^,a^

^a^Department of Bioengineering, University of Illinois at Urbana-Champaign, Urbana, Illinois 61801, USA; E-mail: [rxb@illinois.edu](mailto:rxb@illinois.edu); [dipanjan@illinois.edu](mailto:dipanjan@illinois.edu)

^b^Electrical and Computer Engineering, Chemical and Biomolecular Engineering, Chemistry, and Mechanical Science and Engineering, Beckman Institute for Advanced Science and Technology, University of Illinois at Urbana-Champaign, Urbana, Illinois 61801, USA

^†^These authors contributed equally to this work.


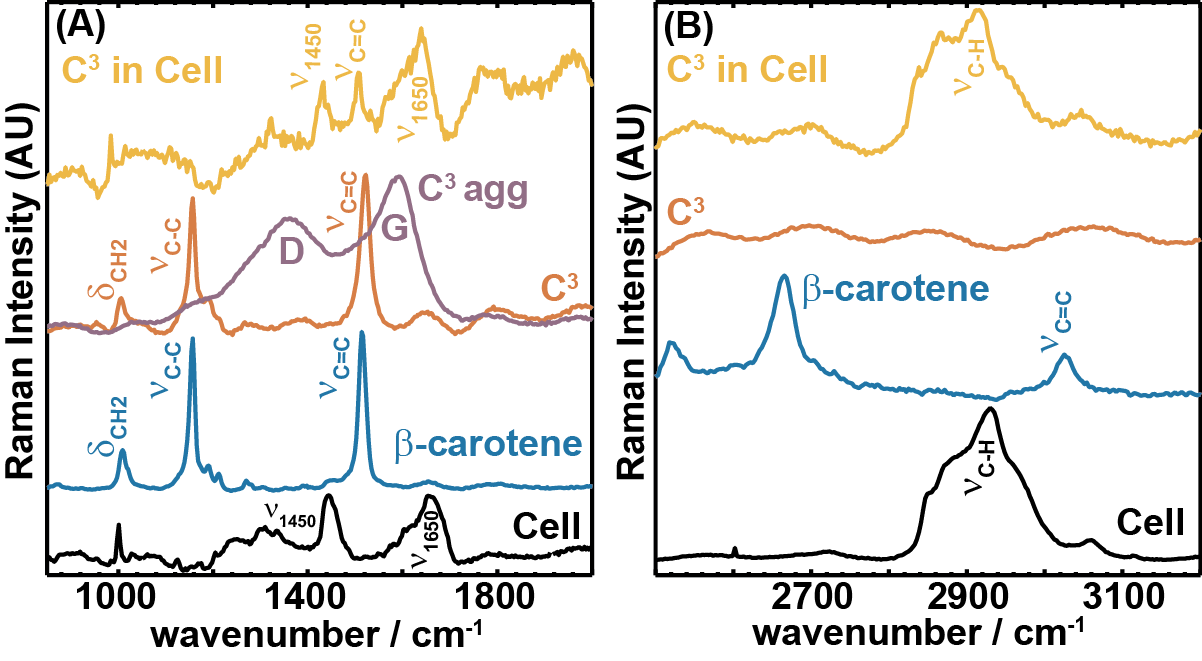


Figure S1. Characteristic Raman spectral features. A and B shows the Raman spectra of cells, b-carotene, C^3^ and a representative dataset for C^3^ incubated in cells at a high concentration. Raman spectra of the Cells by itself have a broad amide I mode with the C-H modes (stretch and bends) much more prominent. Raman spectra of low concentration of C^3^s on the slide match that of the β-carotene, but at high concentrations exhibit features similar to amorphous carbon. The top spectra account for the presence of the C^3^ inside the displaying the traits of C^3^ and cells together.

**Table 1.** Physico-chemical characterization of synthesized NPs. TEM: Transmission electron microscopy; AFM: Atomic force microscopy

**Table 2.** Biological characterization of C^3^-NP and C^3^-Lipocoat nanoparticles.


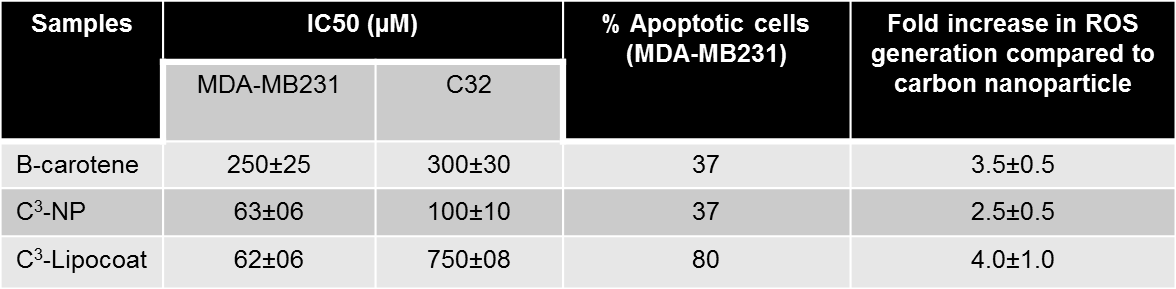


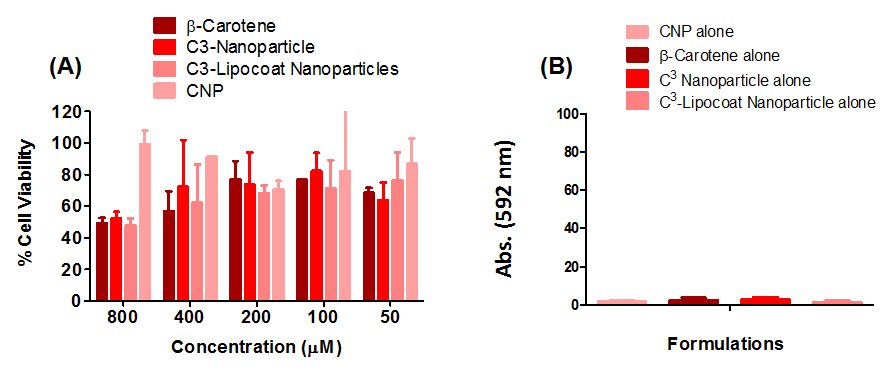


**Figure S2.** (A) MTT assay in MCF-10A cells after 72h of incubation with β-carotene, C3-nanoparticles and C3-Lipocoat nanoparticles at final β-carotene concentration of 50, 100, 200, 400 and 800 µM. CNP alone was used as negative control. (B) Intrinsic absorbance of β-carotene, CNP, C^3^-nanoparticles and C^3^-Lipocoat nanoparticles at λ_592_ nm using amount equivalence of 800 µM. Absorption values from CNP, C^3^-nanoparticles and C^3^-Lipocoat nanoparticles were deducted from the respective results in cell viability studies. β -carotene, C^3^-nanoparticles and C^3^-Lipocoat nanoparticles were dissolved in DMSO to achieve the desired concentration. It presented no significant absorbance value which could affect the final MTT response.
